# Genetic Structure of the Aphid, *Chaetosiphon fragaefolii*, and Its Role as a Vector of the *Strawberry yellow edge virus* to a Native Strawberry, *Fragaria chiloensis* in Chile

**DOI:** 10.1673/031.012.11001

**Published:** 2012-10-03

**Authors:** Blas Lavandero, Pamela Rojas, Claudio C. Ramirez, Marcela Salazar, Peter D.S. Caligari

**Affiliations:** ^1^ Laboratories de Interacciones Insecto-Planta, Instituto de Biología y Biotecnología Vegetal, Universidad de Talca, 2 Norte 685, Talca, Chile; ^2^ Instituto de Biología y Biotecnología Vegetal, Universidad de Talca, 2 Norte 685, Talca, Chile; ^3^ Present address: Instituto de Investigaciones Agropecuarias, Centro Regional Rayentue, Rengo, Chile; ^4^ Present address: Laboratorio de Bioquímica, Departamento de Genética Molecular y Microbiología, Facultad de Ciencias Biológicas, Universidad Católica de Chile, Santiago

**Keywords:** aphid, *Fragaria chiloensis*, genetic structure, microsatellites, virus

## Abstract

The monoecious anholocyclical aphid, *Chaetosiphon fragaefolii* (Cockerell) (Homoptera: Aphididae), was collected on a native strawberry, *Fragaria chiloensis* (L.) Duchesne (Rosales: Rosaceae) from different sites in Chile. The presence of this aphid was recorded during two consecutive years. *F. chiloensis* plants were collected from seven natural and cultivated growing areas in central and southern Chile. Aphids were genotyped by cross-species amplification of four microsatellite loci from other aphid species. In addition, the aphid borne virus *Strawberry mild yellow edge virus* was confirmed in *F. chiloensis* plants by double-antibody sandwich ELISA and RT-PCR. Genetic variability and structure of the aphid populations was assessed from the geo-referenced individuals through AMOVA and a Bayesian assignment test. The presence of *C. fragaefolii*, during the two-year study was detected in only four of the seven sites (Curepto, Contulmo, Chilián and Cucao). Genetic variation among these populations reached 19% of the total variance. When assigning the individuals to groups, these were separated in three genetic clusters geographically disjunct. Of the seven sampled sites, six were positive for the virus by RT-PCR, and five by double-antibody sandwich ELISA . The incidence of the virus ranged from 0–100%. Presence of the virus corresponded with the presence of the aphid in all but two sites (Chilian and Vilches). The greatest incidence of *Strawberry mild yellow edge virus* was related to the abundance of aphids. On the other hand, sequences of the coat protein gene of the different virus samples did not show correspondence with either the genetic groups of the aphids or the sampling sites. The genetic structure of aphids could suggest that dispersal is mainly through human activities, and the spread to natural areas has not yet occurred on a great scale.

## Resumen

El áfido monoico anholocícilico *Chaetosiphon fragaefolii* (Cockerell) (Homoptera: Aphididae) fue colectado sobre *Fragaria chiloensis* en distintas localidades de Chile. Durante dos anos consecutivos se registró su presencia y se tomaron muestras de hojas de *F. chiloensis* en siete localidades, incluyendo sitios naturales y cultivados. Usando amplificación cruzada de partidores microsatelitales de otras especies de áfidos, se genotipificaron los individuos colectados de *C. fragaefolii*. Al mismo tiempo se realizó un ELISA de sandwich confirmación mediante RT-PCR del *Strawberry mild yellow edge virus* de las plantas presentes en las distintas localidades. La variabilidad y estruetura genética de los individuos georeferenciados fue determinada mediante AMOVA y asignamiento Bayesiano. De siete lugares muestreados durante los dos años sólo en cuatro se encontraron ejemplares de *C. fragaefolii* (Curepto, Contulmo, Chilian y Cucao). La variación genética entre localidades fue de un 19 %. Al asignar los individuos a grupos, estos se agruparon en tres unidades genéticas geográficamente separadas. De las siete localidades muestreadas seis localidades fueron positivas para el virus tanto a nivel de ELISA de doble sandwich como de RT-PCR. La incidencia del virus en las distintas localidades muestreadas fluetuó entre 0 y 100 %. La presencia del virus correspondióo con las presencia del áfido en todas las localidades, a excepción de dos de ellas: Chilián y Vilches. La mayor incidencia de *Strawberry mild yellow edge virus* estaría relacionada a una mayor abundancia de áfidos. Por otra parte, las secuencias del gen de la cubierta proteica de las distintas cepas virales no mostraron correspondencia con los grupos genéticos de los áfidos ni con las localidades de colecta. La estruetura genética de los áfidos sugiere que la dispersión ha estado relacionada a las actividades humanas y que invasiones a zonas naturales no han ocurrido aun a una gran escala.

## Introduction

The strawberry aphid *Chaetosiphon fragaefolii* (Cockerell) (Homoptera: Aphididae) is an important pest of strawberry worldwide, presumed to originate from North America. Parthenogenetic forms occur all year round. Although in laboratory cultures and greenhouses male and oviparae female forms occur, this is rarely found in the field (Blackman and Eastop 2000).

In Chile, the presence of this aphid is recent (Zuñiga 1967), and is associated especially with the cultivated strawberry, *Fragaria x ananassa* Duchesne (Rosales: Rosaceae) (Gonzalez 1989), although it has dispersed throughout the whole strawberry production area, including *Fragaria chiloensis* (L.) Duchesne (Rosales: Rosaceae) (Klein-Koch and Waterhouse 2000). *F. chiloensis* is a clonal herbaceous perennial native to grasslands, sand dunes and forests along portions of the Pacific Coast of North and South America. In Chile, its native location, it is distributed from 34° 55′ S to 47° 33′ S (Carrasco et al. 2007), and was traditionally cultivated by the native people before the Spaniards arrived to central Chile in 1542 (Darrow 1966; Wilhelm and Sagen 1972). However, to date the extent to which the aphid *C. fragaefolii* has spread on *F. chiloensis* in Chile has not been studied.

As *C. fragaefolii* persistently transmits several viruses, such as the *Strawberry crinkle virus*, *Strawberry mottle virus, Strawberry mild yellow edge virus* (SMYEV), and *Strawberry vein banding virus* (Krczal 1979, 1982; Blackman and Eastop 2000; Converse 2002; Posthuma et al. 2002), its presence in natural populations might have a negative impact on commercial strawberry production.

Of all of the viruses affecting strawberry, the most common and economically important virus is SMYEV, occurring in both *F. x ananassa* and *F. chiloensis* (Khan 1989). SMYEV is distributed worldwide in cultivated strawberries, and is among the 50 most frequently cited plant diseases in the quarantine regulations of 124 countries (Khan 1989). In this study, the virus was described in detail (Jelkmann et al. 1990), and its full nucleotide sequence obtained (Jelkmann et al. 1991). Nymphs, apterae, and alatae of *C. fragaefolii* all transmit the virus equally well, with 100% transmission occurring with an acquisition feeding period of two days and a transmission feeding period of eight days (Krczal 1979). Although SMYEV has been previously described on *F. chiloensis* (Hepp and Martin 1991), only a small sample was studied, and there is no information on the extent of its spread and incidence, particularly in the natural populations of *F. chiloensis* and the association with the presence of its vector.

In this study, we use heterologous microsatellite markers in order to determine the population structure, diversity, and gene flow of *C. fragaefolii* on wild and cultivated *F. chiloensis* in Chile. The incidence and genetic similarity of SMYEV on different *C. fragaefolii* populations was also assessed.

## Materials and Methods

**Figure 1.**
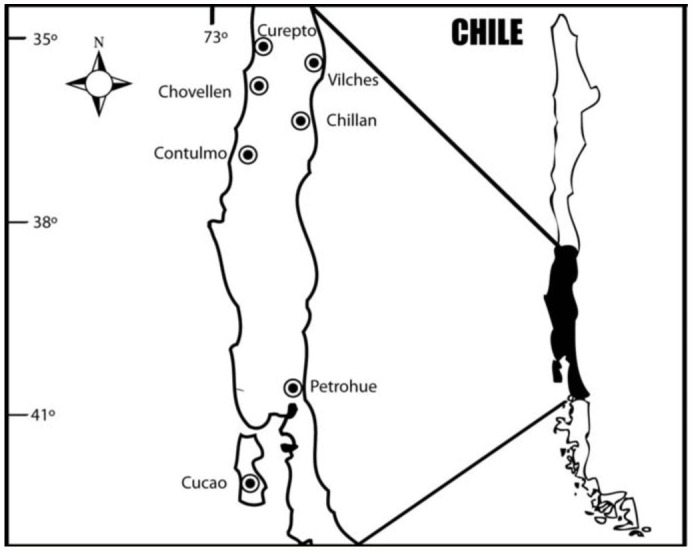
Map of Chile indicating collection sites. High quality figures are available online.

During two consecutive years, seven areas in the central-south of Chile were sampled for *C. fragaefolii* and SMYEV on wild and cultivated *F. chiloensis*. The presence and abundance of aphids was recorded from each site. The sampling sites were Curepto (35° 5′ S, 72° 3′ W), Vilches (35° 36′ S, 71° 12′ W), Chovellen (35° 54′ S, 72° 41′ W), Chilian (36° 35′ S, 72° 4′ W), Contulmo (38° 4′ S, 73° 14′ W), Petrohue (41° 8′ S, 72° 24′ W), and Cucao (42° 35′ S, 71° 7′ W) (Figure 1). When present, 30 to 40 *C. fragaefolii* individuals were collected and kept in 95% alcohol for posterior analysis. To minimize the risk of collecting the same clone, all individuals were collected from different plants separated by least 10 m. Individuals collected were female, wingless adults. Examination under the microscope was done with all individuals to determine species identity. At the same time, 20 to 30 plants were taken per site to assess SMYEV presence and incidence. Plants were kept in aphid-proof cages in a greenhouse, and regular insecticide sprays (Imidacloprid) were done to avoid aphid cross-transmission between plants.

### DNA extraction and polymerase chain reaction amplification for *C. fragaefolii*

Genomic DNA was obtained following the ‘salting out’ procedure from Sunnucks and Hales (1996). The tissue was homogenized with a pestle inside plastic tubes provided with TNES buffer (Tris-HCl 50 mM, pH 7.5, NaCl 400 mM, EDTA 20 mM, SDS 0.5%). The extract was incubated overnight at 37° C with Proteinase K (10 mg mL-1). Proteins were precipitated with NaCl 5M, followed by centrifugation at 10,000 rpm. The supernatant was further washed twice with ethanol under cold conditions, and subjected to centrifugation. The DNA template was suspended in 20 µl of distilled sterile water. Concentration and contamination were assessed with a spectrophotometer.

A total of ten heterologous microsatellite loci were tested, but only five amplified successfully. These were *Sm10, Sm11*, and *Sm17* (Sunnucks et al. 1996), as well as *M62* and *M37* (Sloane et al. 2001). These loci have been shown to successfully amplify in several aphid species (Wilson et al. 2004). Polymerase chain reactions (PCR) were carried out in a Mastercycler® gradient Eppendorf thermocycler (http://www.eppendorf.com), and performed in a 10 µl reaction mixture containing: 1 ng/µl DNA template, 2.5mM MgCl, 0.2 mM dNTP, 0.5 U Taq DNA polymerase (Invitrogen, http://www.invitrogen.com), 0.5 µM of each primer, 20 mM Tris-HCl, pH 8.4, 50 mM KCl. PCR followed a program of 3 min of initial denaturation at 94° C and then 40 cycles of a 1 min denaturation step at 94° C, 1 min of annealing (*Sm10* = 52° C, *Sm11* and *M62* = 55° C, *Sm17* and *M37* = 56.5° C), a 45 sec extension at 72° C, and a final extension at 72° C for 4 min. Amplicons were separated in 6% Polyacrylamide denaturing gels using a BIO-RAD Sequi-Gen GT Electrophoresis Cell. After electrophoresis, gels were silver-stained to visualize the PCR products using the procedure described by Promega (1996). Variation at each locus was recorded by comparing the size of the amplicon in the gel (allele) in base pairs (bp) with the sequence of the PGEM 3ZF(+) vector (Promega Biosciences, http://www.promega.com) loaded in the same gel.

### Detection of SMYEV

Double-antibody sandwich ELISA was performed as described by Clark and Adams (1977) using a commercially available SMYEV IgG and alkaline phosphatase-conjugated SMYEV IgG (BIOREBA, http://www.bioreba.ch). Anti-viral inmunoglobulins were used in polystyrene microtitre plates according to the manufacturer's instructions.

*F. chiloensis* leaf tissue was triturated in 3 ml extraction buffer (20 mM TRIS pH 7.4, 137 mM NaCl, 3mM KCl, 2% PVP, 0.05% Tween 20, 0.02% NaN3) and centrifuged. Aliquots of 100 µl of prepared samples were added to each well duplicated, and negative and positive commercial controls were added to each plate. A total of 20 randomly chosen plants were tested for each site. Samples were read 30 and 60 min after ELISA at 405 nm in a microtitre plate reader VICTOR X3 (PerkinElmer, http://www.perkinelmer.com). ELISA readings were considered positive when the absorbance of sample wells was at least two times greater than the mean absorbance of the negative controls.

As recombinant strains of virus could be undetected using strain specific monoclonal antibodies (Singh et al. 2003), the presence of SMYEV in *F. chiloensis* plants was also confirmed with RT-PCR analysis with coat protein specific primers following the protocol of Thompson et al. (2003). Total RNA of each sample was extracted from 200 mg of leaf tissue, which was homogenized in 2 ml SEB-buffer (0.14 M NaCl, 2 mM Kcl, 2 mM KH_2_PO_4_, 8 mM Na_2_HPO_4_ 2H_2_O (pH 7.4), 0.05% v/v Tween-20, 2% w/v PVP-40, 0.2% w/v ovalbumin, 0.5% w/v bovine serum albumin, 0.05% w/v NaNs) and transferred to a 1.5 mL plastic tube. Then, 100 µl 10% Nlauryl sarcosyl and 5 µl 2-meraptoethanol were added to the tube. From this mixture, a total of 200 µl was taken, and 400 µl of grinding buffer was added and incubated at 70° C with intermittent shaking for 10 min. Then, tubes were placed on ice for 5 min, and centrifuged at 13,000 rpm for 10 min. Next, 150 µl of EtOH, 300 µl 6 M NaI solution, and 25 µl of re-suspended silica were added to the supernatant. Subsequently, the pellet was resuspended in 500 µl of wash buffer and dried. After drying, the pellet was re-suspended in 100 µl of sterilized distilled water, incubated at 70° C for 5 min, and centrifuged at 13,000 rpm for 3 min. RNA integrity was checked by electrophoresis on 1 % agarose gels, and by the A260/A280 ratio using a spectrophotometer (Thermo Scientific NanoDrop, http://www.nanodrop.com).

Complementary DNAs were prepared using SuperScript III Reverse Transcriptase (RT) (Invitrogen) as reported previously by Chang et al. (2007). The RT reaction was carried out with 300 ng of total RNA, 300 ng of random primers, 1 x first-strand buffer, 0.5 mM dNTPs, 10 mM dithiothreitol (DTT), 16 U of RNaseOUT (Invitrogen), and 60 U of SuperScript III RT in a final volume of 50 µl. The reaction was incubated for 2 h at 50° C, and stopped for 10 min at 70° C. For dsRNA templates, a denaturation step using 0.2 mmol of CHsHgOH for 15 min at room temperature was performed prior to the RT reaction. PCR amplifications for virus detection were performed using previously described SMYEV, and internal control AtropaNad2, specific primers (Thompson et al. 2003). PCR products were separated on a 2% agarose gel and visualized under UV light after staining with ethidium bromide.

### SMYEV sequencing

The resulting DNA fragments were cloned in the TOPO® TA vector (Invitrogen). Two µl of the ligation solution was used to transform One Shot Mach1-T1 chemically competent cells (Invitrogen). Plasmids were isolated using QuickClean 5M miniprep kit (GenScript Corp, http://www.genscript.com) from 3 ml overnight cultures containing ampicillin. Recombinant plasmids were verified by EcoR1 digestion. Sequencing reactions were performed at the Macrogen Inc. facilities (http://www.macrogen.com) in an ABI3730 XL automatic DNA sequencer.

**Table 1.**
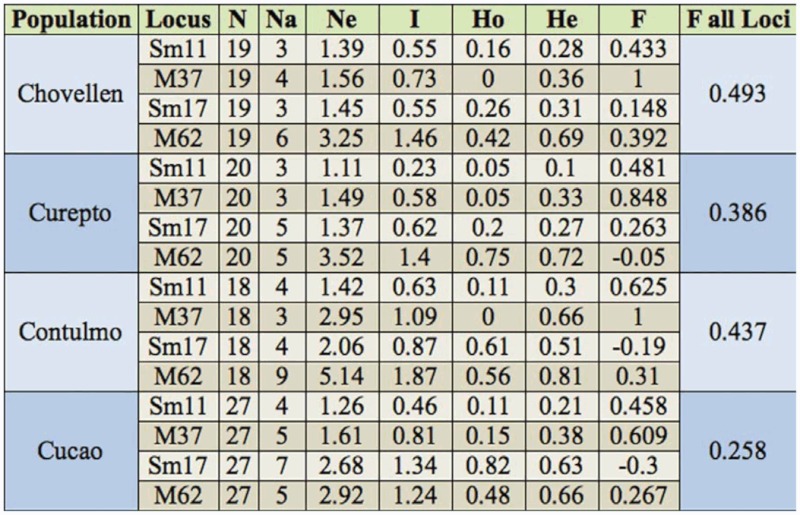
Sample sizes (N), Number of alleles (Na), Number of effective alleles (Ne), Information index (l), Observed and Expected Heterozygosity (Ho and He), Fixation Index (F) per locus per population and F per population (all loci).

### Aphid population structure

A total of five loci were considered (Table 1), but as locus *Sm10* was invariant for most populations, only four loci were considered for the final analysis. Results were first analyzed using Micro-Checker 2.2.3 to check microsatellite data for null alleles and scoring errors. Fstat (Goudet 2002) was used to calculate observed and expected heterozygosity, as well as linkage disequilibrium between loci. An exact test was used to detect significant deviations from Hardy-Weinberg equilibrium (HWE) using Arlequin 3.11 (Excoffier et al. 2005). Sample sizes, number of alleles, effective number of alleles, information index, observed and expected heterozygosity, fixation index per locus per population and fixation index per population (all loci) were calculated with Genalex 6 (Peakall and Smouse 2006). The use of different methods to study the spatial genetic structure of organisms in a sampled region has been strongly recommended (Frantz et al. 2006; Pearse and Crandall 2005; Storfer et al. 2007); therefore, structure was assessed using two methods. First, a molecular analysis of variance (AMOVA) was carried out, and pairwise values between collection sites were estimated using Genalex 6 (Peakall and Smouse 2006). The proportion of the variance among populations relative to the total variance was estimated considering genotypic information (*PhiPT*). *PhiPT* is analogous to *Fst* when the data are haploid, or when assumptions of HWE are not met (Maguire et al. 2002). Isolation by distance was checked using a Mantel test between genotypic differentiation (*PhiPT*) and the geographical distance between sites, using zt version 1.0 with 10,000 permutations (Bonnet and Van de Peer 2002). The second method for assessing the populations structure was a Bayesian clustering method described by Corander et al. (2003), implemented in software BAPS version 4.14 (Corander et al. 2006). This was used to determine the genetic structure of *C. fragaefolii*. This software uses stochastic optimization to infer the genetic structure, and it can use a spatial model that takes into account individual geo-referenced multilocus genotypes to assign the biologically relevant structure, thereby increasing the power to detect correctly the underlying population structure (Corander et al. 2006). To run the program, a number K of genetic clusters characterized by the matrices of allele frequencies at each locus is first assumed. Then, for each individual, the proportion of its genome derived from each genetic cluster (proportion of ancestry) is estimated. The posterior probability (probability of K given the data) is then calculated for each mean value of K using the mean estimated log-likelihood of K to choose the optimal K. Ten independent repetitions for each K from 1 to 4 were carried out following the recommendations of Corander et al. (2003).

**Table 2.**
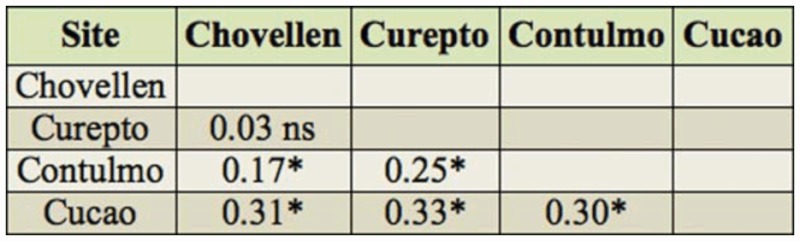
*PhiPT* values between sites; * significant values after 1000 permutations.

#### Genetic similarity and divergence between SMYEV sequences

In order to compare the genetic similarity and the divergence pattern between SMYEV sequences of sites where positive identification occurred, samples were aligned with ClustalX version 2 (Larkin et al. 2007), and their phylogenetic relationships were inferred using the neighbor-joining method implemented in MEGA4 (Tamura et al. 2007). The evolutionary distances were computed using the maximum composite likelihood method (Tamura et al. 2004) and bootstrap with 10,000 replicates.

### Results

#### Aphid genetic variability and structure

**Figure 2.**
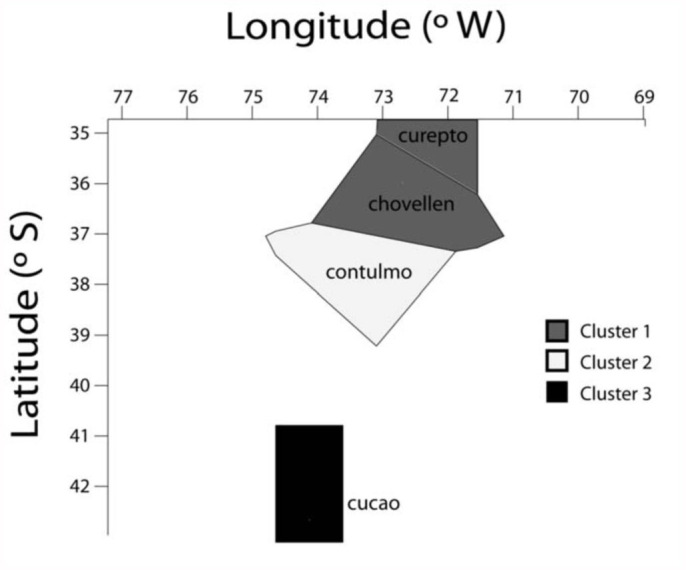
Voronoi tessellations of the spatial clustering of individuals from four sitess. A cell of the tessellation corresponds to the physical neighborhood of an observed data point, and is colored according to the cluster membership. High quality figures are available online.

As derived from Table 1, mean observed heterozygosity across loci of *C. fragaefolii* populations varied from 0.21 to 0.38, being highest at Cucao (0.38) and Contulmo (0.31). The number of effective alleles varied from 3 and 9 (Table 1). All loci departed significantly from HWE, with the exception of *Sm17* in Cucao and Chovellen. While no linkage disequilibrium was evident between loci, frequency of null alleles using the EM algorithm (Dempster et al. 1977) implemented in software FreeNA (Chapuis and Estoup 2007) was high for locus *M37* (0.19 across populations). Therefore HWE was estimated excluding *M37*, with no significant difference with the estimations including *M37*. Genetic differentiation in the data was modest, with *PhiPT* values reaching 0.19, although *PhiPT* values between pairs of sites varied from 0.03 to 0.33 (Table 2). The greatest genetic difference occurred between Cucao and the other sites (Table 2). There was significant isolation by distance as evidenced by the Mantel test (r = 0.94; *p* = 0.008). The number of clusters in optimal partition assignment with BAPS determined K = 3, with a log marginal likelihood of optimal partition of 757.4084, and the posterior probability reaching its highest value (∼1). Aphids from sites Chovellen and Curepto conformed one cluster, whereas aphids from Contulmo alone formed a second cluster. Aphids from Cucao formed a third cluster (Figure 2). This same grouping was observed when considering the site of individuals and their spatial coordinates in the model.

#### Presence and abundance of *C. fragaefolii* and incidence of SMYEV

**Figure 3.**
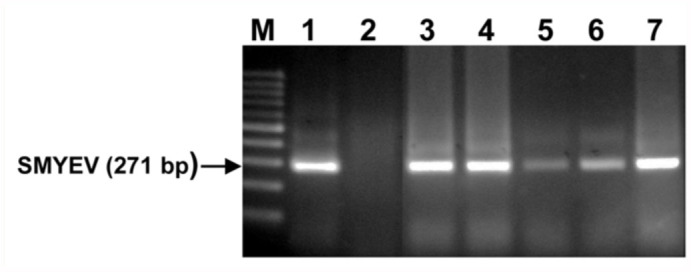
RT-PCR of *Fragaria chiloensis* isolates indicating the presence of SMYEV. Lanes 1–7: Cucao, Petrohue, Contulmo, Curepto, Chilian, Vilches and Chovellen. M = 100 bp molecular weight marker. *SMYEV* = *Strawberry mild yellow edge virus*. High quality figures are available online.

From the seven sites sampled during 2005 and 2006, only four had the aphid (*C. fragaefolii*). SMYEV, however, was detected at six of these seven sites. Mean aphid abundance per leaf per site ranged from 0.8 to 14.5, with the highest numbers occurring at Contulmo (Table 3). SMYEV incidence revealed by ELISA was highest at Contulmo (Table 3). All strawberry plants of the seven sites tested by RT-PCR with the capsid primer of SMYEV revealed amplification, with the exception of Petrohue (Figure 3).

**Table 3.**
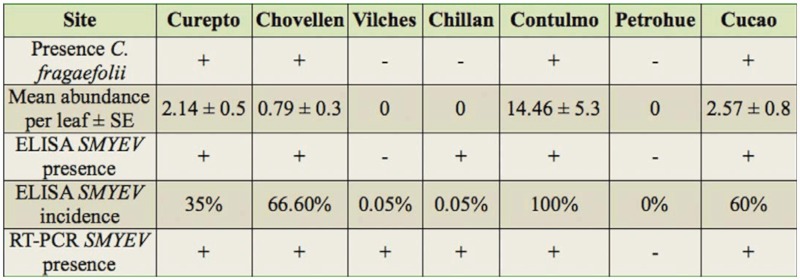
Presence of *Chaetosiphon fragaefolii*, mean abundance ± SE, presence and incidence of *SMYEV* using double-antibody sandwich ELISA and RT-PCR at the collection sites. *SMYEV* = *Strawberry mild yellow edge virus*

**Figure 4.**
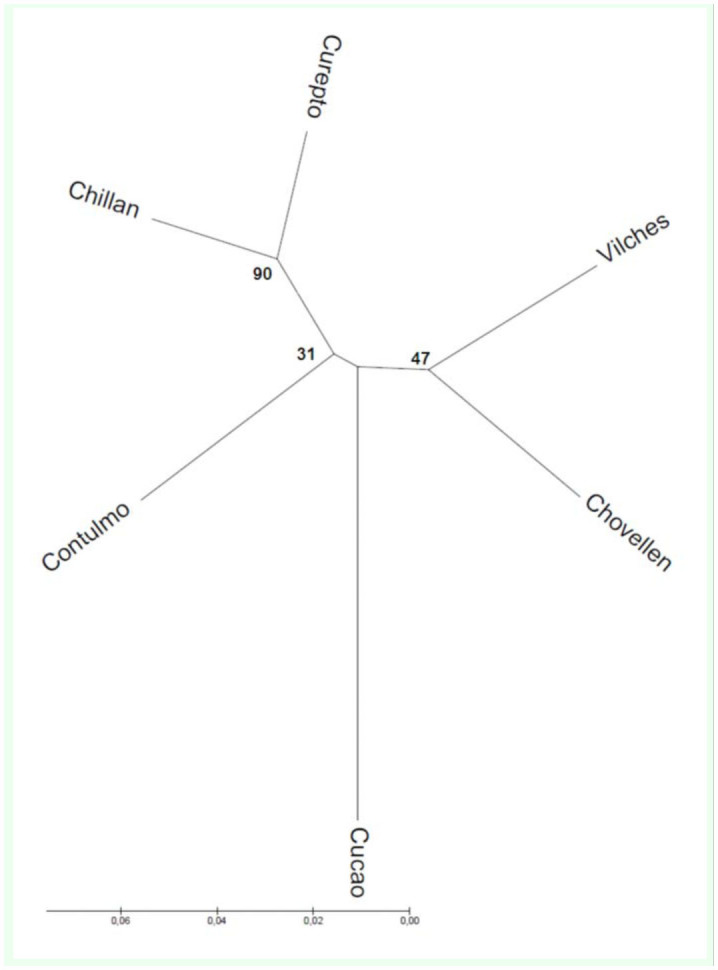
Unrooted tree describing the phylogenetic relationships of SMYEV inferred using the neighbor-joining method. The optimal tree with the sum of branch length = 0.27935868 is shown. The tree is drawn to scale, with branch lengths in the same units as those of the evolutionary distances used to infer the phylogenetic tree. *SMYEV* = *Strawberry mild yellow edge virus*. High quality figures are available online.

The phylogenetic relationship among SMYEV sequences revealed a larger genetic distance of Cucao (south) when compared with the remaining samples (Figure 4). In addition, among the northern populations, Contulmo formed a separated lineage from Chovellen and Curepto, while these two were found to belong to different branches in the unrooted tree (Figure 4).

### Discussion

Observed heterozygosity of *C. fragaefolii* populations was highest at Cucao and Contulmo. Such values are lower than those reported in studies from other aphid species (Hales et al. 1997; Figueroa et al. 2005; Lavandero et al. 2011), which may be explained by the absence of sexual reproduction of this species, and the recent introduction of only a few clones (Blackman and Eastop 2000). Similarly, significant departures from HWE were also expected because parthenogenesis is the only mode of reproduction reported for this species (Blackman and Eastop 2000). However, one locus (*M37*) presented high values of null alleles, but was consistent over all populations, and did not affect the overall results. Genetic variability was modest, showing geographic structure especially with the most southern population (Cucao). Bayesian analysis showed three clear clusters, which was in agreement with the AMOVA results, as sites in each cluster shared low *PhiPT* values, and the highest *PhiPT* values were between sites that were assigned to other clusters (Table 2, Figure 2), confirming that *C fragaefolii* populations on *F. chiloensis* consist of these distinct clusters.

Clusters agreed with geographical distance between sampling sites, as areas that were close to each other formed a single cluster, and sites further away formed separated genetic clusters (Figure 2). In fact this was confirmed by the Mantel test, showing significant isolation by distance. This may be related to the fact that *F. chiloensis* has a fragmented distribution across the studied area, making aphid dispersal difficult between sites. Indeed, the low gene flow of *C. fragaefolii* between geographical areas, with exception of the nearby sites (Curepto and Chovellen), suggests little migration between sites. It is important to mention that Cucao is a population located on the Chiloé island, a rather big island in the south of Chile. This may explain the genetic distance of the southern *C. fragaefolii* from the northern populations.

The phylogenetic relationship of SMYEV showed Cucao as a genetically distant group from the northern samples, resembling the higher genetic differentiation exhibited by the aphid *C. fragaefolii* on *F. chiloensis* (cluster 3, Figure 2). It is worth noting that the remaining SMYEV sequences were not clearly different. Thus, with the exception of the Cucao sample, SMYEV sequence divergence does not fully agree with the genetic structure of its aphid vector, suggesting that the virus was present before the arrival of the aphids. As mentioned above for the case of *C. fragaefolii*, the fact that the Cucao population is located on an island may also explain the divergence from the northern samples of SMYEV.

The absence of *C. fragaefolii* at one site (Petrohue, Table 3) was coincident with the absence of the vector. At the other two sites where *C. fragaefolii* was not found (Vilches and Chillán), the virus had a low incidence (0.05%). At both sites, however, *Neuquenaphis edwardsi* (Laing) (Hemiptera: Aphididae), a native species of aphid frequently found on southern beech Nothofagus sp. Blume (Fagales: Nothofagaceae), was found developing on wild *F. chiloensis*. It is common to find (as is the case for both sites) *F. chiloensis* associated to *Nothofagus* forests (San Martin et al. 1991). The aphid species could act as a vector between plants, perhaps in a non persistent way. Although SMYEV has been shown to be mainly transmitted by *C. fragaefolii*, other species of aphids have also been shown to effectively infect strawberry (Converse 1987). Therefore, the capacity to vector the virus may be very restricted, this restriction being reflected in the low incidence values.

The fact that the virus was found in a native aphid species may complicate the spread of the virus further, but it is still not clear whether it is the same strain, as preliminary results of the sequence of the virus present in *N. edwardsi* suggest variation in size of the virus capsid RT-PCR products (Salazar, unpublished data). Further studies are needed to better understand these findings and to confirm *F. chiloensis* as an alternative host for *N. edwardsi*, or for other native aphid species. It has been reported that introduced aphids can be very harmful to the native flora (Malmstrom et al. 2005), particularly in island ecosystems where aphid species with permanent parthenogenesis are more likely to develop successful colonies, and therefore be more efficient in virus transmission (Mondor et al. 2007). On the other hand, the highest SMYEV incidence values are associated with the highest abundance of aphids in the greatest *F. chiloensis* cultivation area (Contulmo) (Carrasco et al. 2007). Most orchards here also cultivated, *F*. x *ananassa*, with little control measures of *F. chiloensis*.

As *C. fragaefolii* aphids have been introduced and spread recently, the low incidence of the virus in the native *F. chiloensis* could be explained. However, sequence data of the virus found on *F. chiloensis* suggest that the virus is not different between cultivated and wild *F. chiloensis*. Thus, the presence of the aphid clearly increases the incidence of the virus, even though it is already present in natural and cultivated *F. chiloensis* with no clear grouping of the viral strains, with the exception of Cucao. Cucao is the most southern group, and phylogenic trees could suggest isolation.

## Abbreviations

HWE: Hardy—Weinberg equilibrium
RT-PCR: reverse transcriptase PCR
SMYEV: *Strawberry Mild Yellow Edge Virus*
